# Energy drink consumption in a pluri-ethnic population of adolescents in the Pacific

**DOI:** 10.1371/journal.pone.0214420

**Published:** 2019-03-22

**Authors:** Stéphane Frayon, Guillaume Wattelez, Sophie Cherrier, Yolande Cavaloc, Yannick Lerrant, Olivier Galy

**Affiliations:** Interdisciplinary Laboratory for Research in Education, School of Education, University of New Caledonia, Av James Cook, Nouméa Cedex, New Caledonia; University 9 of July, BRAZIL

## Abstract

**Objective:**

Energy drinks are very popular among teenagers but may cause health problems. Energy drink consumption is partly associated with energy drink perception, but little is known about this in the Pacific Island Countries and Territories. The aim of this cross-sectional study was to identify the relationships between energy drink consumption, energy drink perception, weight status and sociodemographic characteristics in a school-going sample of Pacific adolescents.

**Design:**

A cross-sectional study carried out in the schools during school hours between July 2015 and April 2016.

**Setting:**

Sociodemographic characteristics, weight status, energy drink perception, and quantity of energy drinks consumed were obtained. Chi-square tests of independence, independent *t* tests, multivariate logistic regressions and multiple linear regressions were used.

**Subjects:**

A representative sample of 678 New Caledonian adolescents (11–16 years).

**Results:**

We found that one third of New Caledonian adolescents consume energy drinks. Boys are more likely to drink them than girls and Polynesians drink significantly more than European and Melanesian adolescents. Higher energy drink consumption in the New Caledonian adolescents is associated with good or neutral perceptions of the energy drink impact on health. Moreover, sex (being male) significantly influences the total energy drink consumption per week. Energy drink consumers have a tendency toward better perceptions of energy drinks than non-consumers.

**Conclusions:**

Nutritional education targeting energy drink consumers should take these results into account by providing (community-based) educational programs, especially for adolescents from low socioeconomic backgrounds, boys, or those living in rural areas.

## Introduction

Energy drinks (EDs) are non-alcoholic beverages containing caffeine, sugar and other stimulant ingredients. According to self-report surveys, 30% to 50% of adolescents and young adults consume EDs [1]. In a European study, the biggest ED consumers were young people between 10 and 18 years old (68%), followed by adults over 18 years (30%) and children under 10 years (18%) [2].

The caffeine in EDs can decrease the duration and quality of sleep and may even cause caffeine intoxication [1]. Energy drink consumption (EDC) can lead to excessive sugar intake and may cause long-term health problems, such as dental erosion, obesity and type 2 diabetes [3]. A positive association between EDC and symptoms of mental health problems [4,5] and risky behaviors (binge drinking, smoking, engagement in unsafe sex, violent behaviors, risky motor vehicle use and disordered eating behaviors) [5,6] has been established. Yet despite these findings, ED perception is often positive in adolescent populations [7] and this positive perception has been associated with higher EDC [8].

Little is known about EDC and ED perceptions in the Pacific Island Countries and Territories (PICT). Most of the studies in the Pacific area have been conducted in Australia, where 42 to 48% of the youth consume them [6, 9]. In New Zealand, one third of the adolescents reported consuming EDs in the previous week [9]. Moreover, in this study Utter showed that EDC was linked with ethnicity and socioeconomic status (SES), with high EDC being more common among Pacific Island students living in high-deprivation areas [9]. However, the relationship between ED perception and EDC in adolescents living in PICT had never been studied to our knowledge.

New Caledonia, a French archipelago in the South Pacific, is characterized by high diversity in ethnicity and SES. The prevalence of obesity in adults and adolescents is high [10,11], as is the prevalence of diabetes in adults [12]. In school-going adolescents, high sugar-sweetened beverage consumption [13] and the high prevalence of dental concerns have been described [14]. In this context, high EDC might exacerbate these health concerns. Moreover, the high EDC observed in Pacific Islanders, especially the low-income adolescents in the region [9], suggests that sociocultural background may influence EDC. To our knowledge, however, no study has specifically examined EDC and perceptions in New Caledonian adolescents. To address this gap in the literature and inform public health efforts targeting consumption, this study used data from a population-based survey in order to describe EDC among New Caledonian adolescents and examine the associations with SES, anthropometric characteristics and ED perceptions. We hypothesized that (1) EDC would be associated with perceptions about EDs in New Caledonian adolescents and (2) ED perception would be associated with sociodemographic factors (SES, ethnicity, area of residence and sex) and/or weight status.

## Materials and methods

This cross-sectional study was conducted with school-going adolescents (11–16 years old) between July 2015 and April 2016. The study was carried out in the schools during school hours.

### Population

New Caledonia is divided into three provinces with marked differences in ethnic composition, SES, and urbanization. Five secondary public schools were randomly selected: one in Loyalty Islands Province (rural area), two in North Province (east and west coasts, rural areas) and two in the capital of South Province, the only urban zone of New Caledonia. Two classes were then selected in each of four grades (levels) by a staff member, for a total of 160 students (8 groups with a mean of 20 students per division). Adolescents absent or with missing data (including parental refusal) (14.0%) and those from ethnic groups other than Melanesian, European or Polynesian (1.5%) were excluded. The final number of participants was 678, with ages ranging from 11 to 16 years old.

Parents gave informed written consent prior to their children’s participation in the study. The study protocol was approved by the ethics committee of the University of New Caledonia. The protocol also met all legal requirements and the criteria of the Declaration of Helsinki.

### Sociodemographic information

The demographic information used in the analyses included age, sex, ethnicity, SES and area of residence. Ethnicity was self-reported by the adolescents and categorized as recommended in the INSERM report on New Caledonia using an anonymous survey tool [15]. Birthdates, sex and SES were collected in the school databases. SES was indexed from the occupation of the household reference person (defined as the householder with the highest SES after coding) using the National Statistics Socio-Economic Classification. For the present analyses, we generated three categories: managerial and professional occupations (high SES), intermediate occupations (intermediate SES), and routine and manual occupations (low SES). The degree of urbanization was determined using a European standard [16] and the data of the last census in New Caledonia [17]. Densely populated areas comprising at least 50,000 inhabitants in a continuous zone with more than 500 inhabitants per km^2^ were classified as urban. Rural areas were defined as areas with fewer than 50,000 inhabitants and fewer than 100 inhabitants per km^2^, and not adjacent to an urban area.

### Energy drink consumption and opinion

EDC was assessed with several questions. The first one was: “Do you drink energy beverages?” For non-consumers, the reasons for non-consumption were explored by the question: "You never drink energy drinks because . . . ." The multiple response options were don't like, parental refusal, medical refusal or other. For consumers, the average EDC was assessed with the question: “How many cans of Red Bull/Lift (250 mL), Monster (473 mL), or others of this type (250 mL) do you consume per week on average?” Examples of popular brand names in New Caledonia were provided to allow the participants to distinguish EDs from soft/sports drinks. The total ED intake per week, converted to milliliters, was calculated by adding up the volumes of the total number of cans that were consumed, and the adolescents who answered “No” to the first question were considered as consuming 0 L per week. To allow comparison with other studies, EDC was also converted to the usual categories of: 1 can/week, 2–4 cans/week, 5–6 cans per week, 1 can/day, 1–2 cans/day, and more than 2 cans/day. In this case, can volume (250 or 473 mL) was not taken into account. All the participants were asked to give an opinion on EDs by answering the question: “Do you think that energy drinks are…?” The response options were good for health, rather good for health, with no effect on health, rather bad for health, and bad for health. Three categories of responses were created for this variable: good (good/rather good), neutral (no effect) and bad (bad/rather bad).

### Anthropometric parameters

Anthropometric parameters (height and weight) were collected by trained staff in the school nurse’s office, as previously described [10]. Body mass index (BMI) was calculated by dividing weight in kilograms by height squared in meters. As BMI values to define overweight vary with age and sex in adolescents, we calculated the BMI z-score and percentile for each participant using the reference values of the International Obesity Task Force (IOTF) [18]. Weight status was defined according to the IOTF criteria [18]. Thus BMI z-score values of more than 1.310 and more than 1.244 were used to define overweight boys and girls, respectively, corresponding to a BMI value of 25 in adults according to Cole et al. [18].

### Data analysis

All analyses were conducted using SPSS version 22.0, with the significance level set at *P* < .05. As previous studies found that EDC was higher in boys than girls [19], we analyzed data by sex. The sex and ED consumer/non-consumer differences were assessed using t-tests (continuous variables) or χ^2^ tests (categorical variables).

We used multivariate logistic regressions to identify the factors associated independently with EDC. The variables in the models were age (in years as a continuous variable), sex (boys vs. girls), ethnicity (Caucasian, Melanesian or Polynesian), SES (high, intermediate or low), area of residence (urban vs. rural) and weight status (overweight or not). Similar analyses were conducted to identify the factors associated with poor or good opinions about EDs using the same variables.

Multiple linear regression analysis identified the factors associated with the total amount of EDC. The variables in the models were age, sex, ethnicity, SES and weight status (overweight or not). Categorical variables were categorized into groups by creating dummy variables.

## Results

The sample included 314 boys and 364 girls from 11 to 16 years old (13.6±1.5 years). Out of the whole sample, 42.3% declared that they drank EDs. Table 1 shows the overall descriptive data for ED consumers and non-consumers. The results show that the distribution between them differed with sex, ethnicity, SES, area of residence, and perception of ED, but not with weight status.

**Table 1:** Sociodemographic characteristics and perceptions of energy drinks in energy drink users and non-users.

	ED Non-users	ED Users	P*
	(n = 391)	(n = 287)	
Age (years)	13.63±1.47	13.57±1.42	.635
Sex % (n)			
Male	51.6 (162)	48.4 (152)	.003
Female	62.9 (229)	37.1 (135)	
Ethnicity % (n)			
European	66.2 (151)	33.8 (77)	.004
Melanesian	52.8 (218)	47.2 (195)	
Polynesian	59.5 (22)	40.5 (15)	
SES % (n)			
High	70.5 (148)	29.5 (62)	< .001
Intermediate	57.4 (97)	42.6 (72)	
Low	48.8 (146)	51.2 (153)	
Residence % (n)			
Urban	70.2 (125)	29.8 (53)	< .001
Rural	53.2 (266)	46.8 (234)	
Perception of ED % (n)			
Bad for health	67.1 (347)	32.9 (170)	< .001
Neutral	29.7 (22)	70.3 (52)	
Good for health	25.3 (22)	74.7 (65)	
Overweight % (n)			
Yes	55.3 (131)	44.7 (106)	.355
No	59.0 (260)	41.0 (181)	

Mean ± SD or %

ED = energy drinks; SES = socioeconomic status

*P values are for the association between each variable and ED consumption

We examined the reason why some adolescents choose not to drink EDs. Most of them (60.6%) declared that they disliked EDs. The second reason was mainly parental refusal of consumption (35.5%), while the other reasons were very infrequent (medical refusal: 5.5% and other reasons: <1%). In a multivariate analysis, we then examined the factors associated with each of these reasons. No specific profiles emerged concerning adolescents who declared that they disliked EDs. However, we found that adolescents with high SES were more likely to cite parental refusal of EDC than those with low SES (high SES: OR = 0.546;P = .026). Parental refusal of consuming ED was also negatively associated with age (OR = 0.798;P = 0.004) but not with weight status, ethnicity or area of residence.

The factors independently associated with EDC were determined by multivariate analysis. Results are shown in Table 2. Adolescents who thought that EDs are good for or without effect on health were more likely to consume these beverages than those who thought they had a bad effect on health (Good for health: OR = 5.119; *P* < .001; neutral: OR = 4.247 *P* < .001). Boys were more likely to consume EDs than girls (OR = 1.707; *P* = .002). Low SES girls were more likely than high SES girls to declare that they consumed EDs (OR = 2.588; *P* = .005). Area of residence (urban or rural), weight status and ethnicity were not independently associated with EDC.

**Table 2:** Predictors of energy drink consumption (multivariate analysis$).

	Whole sample (n = 678)			Boys (n = 314)			Girls (n = 364)		
	%* (n)	OR [95% CI]	*P*	%* (n)	OR [95% CI]	*P*	%* (n)	OR [95% CI]	*P*
Age (years)†		1.070 [0.946–1.211]	.281		1.291 [1.083–1.539]	.004		0.866 [0.718–1.044]	.866
Sex									
Female	37.1 (135)	1.00							
Male	48.4 (152)	1.707 [1.221–2.386]	.002						
Ethnicity									
European	33.8 (77)	1.00		39.4 (41)	1.00		29.0 (36)	1.00	
Melanesian	47.2 (195)	1.066 [0.718–1.582]	.751	52.4 (100)	1.051 [0.585–1.885]	.868	42.8 (95)	1.124 [0.650–1.945]	.676
Polynesian	40.5 (15)	1.501 [0.687–3.277]	.308	57.9 (11)	2.749 [0.912–8.282]	.072	22.2 (4)	0.749 [0.210–2.668]	.656
SES									
High	29.5 (62)	1.00		39.6 (40)	1.00		20.2 (22)	1.00	
Intermediate	42.6 (72)	1.306 [0.820–2.078]	.261	48.3 (42)	1.159 [0.598–2.244]	.663	36.6 (30)	1.667 [0.835–3.330]	.148
Low	51.2 (153)	1.807 [1.171–2.788]	.008	55.6 (70)	1.318 [0.688–2.526]	.406	48.0 (83)	2.324 [1.260–4.290]	.007
Residence									
Urban	29.8 (53)	1.00		37.4 (34)	1.00		21.8 (19)	1.00	
Rural	46.8 (234)	1.498 [0.939–2.390]	.090	52.9 (118)	1.865 [0.973–3.575]	.060	41.9 (116)	1.121 [0.544–2.308]	.757
Energy drink perception									
Bad for health	32.9 (170)	1.00		37.8 (88)	1.00		28.9 (82)	1.00	
No effect on health	70.3 (52)	4.247 [3.003–8.127]	< .001	74.4 (29)	3.920 [1.772–8.673]	< .001	65.7 (23)	4.052 [1.873–8.766]	< .001
Good for health	74.7 (65)	5.119 [2.452–7.355]	< .001	83.3 (35)	7.582 [3.151–18.243]	< .001	66.7 (30)	3.884 [1.936–7.794	< .001
Weight status									
Overweight	44.7 (106)	1.00		50.0 (52)	1.00		40.6 (54)	1.00	
Not overweight	41.0 (181)	0.893 [0.625–1.277]	.537	47.6 (100)	0.969 [0.567–1.656]	.909	35.1 (81)	0.804 [0.492–1.314]	.384

OR: odds ratio; CI: confidence interval; SES: socioeconomic status

*Indicates the percentage of adolescents in each group consuming energy drinks.

†Entered into the model as a continuous variable.

$Variables in the models are: age (years), sex, ethnicity, SES, residence, energy drink perception and weight status category.

**Fig 1:**
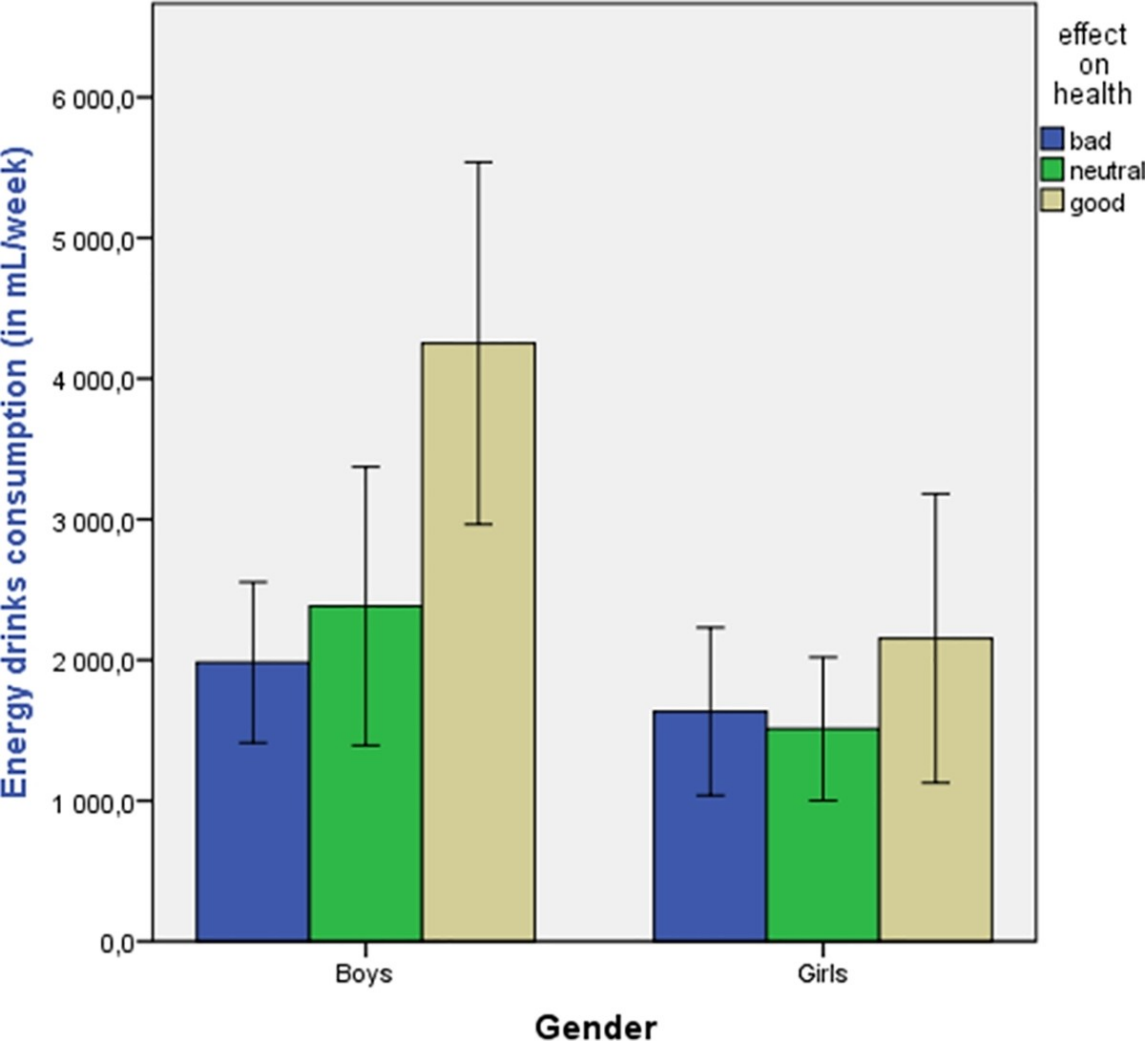
Energy drink consumption per week stratified by sex and opinion about effects on health.

In the ED consumer subgroup (n = 237), participants declared consuming on average 2130 mL/week (304 mL/day). Fig 1 shows the EDC per week according to sex and opinion about ED effects on health. We found that EDC was twice as high in boys who reported that EDs are good for health (4118 mL/week) than in boys (1922 mL/week) and girls (1602 mL/week) reporting that EDs are bad for health. In the ED consumer subgroup, 26.8% drank <2 cans/week, 29.6%: 2–4 cans/week, 15.3%: 4–7 cans/week, 16.7%: ≥1 and <2 cans/day, and 11.5%: ≥2 cans/day.

The multiple regression model (Table 3) of the ED consumer subgroup indicated that sex (being male, β = 0.165; *P* = .005) and living in a rural area (β = 0.158; *P* = .034) were positively associated with higher EDC. Moreover, the total amount of EDC was positively associated with positive opinions about ED effects on health (β = 0.172; *P* = .005). Other factors (age, SES, ethnicity, area of residence or weight status) were not associated with the total quantity of EDC.

**Table 3:** Linear regression models to examine predictors of amount of energy drink consumption.

Variable	(Adjusted R^2^ = 0.092, F = 3.80 (df = 10), *P*<0.0001)	
	β (95% CI)	*P* value
Age (years)	-0.103 (-0.228–0.023)	.108
Boys†	0.165 (0.051–0.278)	.005
Melanesian‡	0.093(-0.039–0.225)	.168
Polynesian‡	0.087 (-0.036–0.209)	.164
Rural$	0.158 (0.012–0.305)	.034
Low SES§	-0.045 (-0.207–0.116)	.582
Inter SES§	-0.011 (-0.159–0.138)	.889
Good for health*	0.172 (0.052–0.292)	.005
No effect on health*	0.0079 (-0.110–0.125)	.901
Overweight¥	0.013 (-0.100–0.126)	.825

Reference is:

† girls

‡ European

$urban

§high SES

*bad for health

¥not overweight

We found that ED consumption and the amount of consumption were associated with opinions about the effects of ED on health, and we therefore used multivariate analysis to investigate the factors associated with poor or good opinions about ED (Table 4). ED non-consumers were more likely to have a poor opinion of EDs than consumers (OR = 4.711; *P* < .001). Melanesian adolescents were more likely to think that ED are good for health than Europeans (OR = 1.950; p = .042). Similarly, adolescents with high SES, overweight or living in urban areas were more likely to think that ED have a bad effect on health (Table 4). Age and sex were not independently associated with opinions about ED effects on health.

**Table 4:** Predictors of good and bad opinion of energy drinks (multivariate analysis$).

	Whole sample (n = 678)					
	Bad opinion (n = 517)			Good opinion (n = 87)		
	%* (n)	OR [95% CI]	*P*	%* (n)	OR [95% CI]	*P*
Age (years)†		0.899 [0.771–1.049]	.176		0.955 [0.792–1.152]	.630
Sex						
Girls	78.0 (284)	1.00		12.4 (45)	1.00	
Boys	74.2 (233)	0.905 [0.690–1.344]	.620	13.4 (42)	1.001 [0.618–1.623]	.996
Ethnicity						
European	86.4 (197)	1.00		6.1 (14)	1.00	
Melanesian	69.5 (287)	0.532 [0.326–0.870]	.012	16.9 (70)	1.951 [1.024–3.716]	.042
Polynesian	89.2 (33)	0.986 [0.301–3.224]	.981	8.1 (3)	1.721 [0.427–6.926]	.445
SES						
Low	69.2 (207)	1.00		18.4 (55)	1.00	
Intermediate	72.8 (123)	0.910 [0.570–1.452]	.691	12.4 (21)	0.821 [0.461–1.463]	.503
High	89.0 (187)	2.038 [1.160–3.580]	.013	5.2 (11)	0.821 [0.224–0.963]	.039
Residence						
Urban	89.9 (160)	1.00		4.5 (8)	1.00	
Rural	71.4 (357)	0.441 [0.229–0.847]	.014	15.8 (79)	2.029 [0.841–4.898]	.116
Weight status						
Overweight	78.5 (186)	1.00		12.2 (29)	1.00	
Not overweight	75.1 (331)	0.610 [0.401–0.929]	.021	13.2 (58)	1.405 [0.847–2.330]	.188
ED consumption						
Yes	59.2 (170)	1.00		22.6 (65)	1.00	
No	88.7 (347)	4.711 [3.126–7.100]	< .001	5.6 (22)	0.249 [0.147–0.422]	< .001

OR: odds ratio; CI: confidence interval; SES: socioeconomic status

*Indicates the percentage of adolescents in each group with a bad or good opinion about energy drink effects on health.

†Entered into the model as a continuous variable.

$Variables in the models are: age (years), sex, ethnicity, SES, residence, energy drink consumption and weight status category.

## Discussion

More than one third of New Caledonian adolescents consume EDs. This study also shows that adolescents with good or neutral perceptions of the impact of EDs on health are more likely to consume this type of beverage and drink them in greater quantities than those with negative perceptions. This perceptions of adolescents about the ED impact on health were correlated with ethnicity, area of residence, SES and ED consumption.

The proportion of adolescents consuming ED (42%) is very close to that found by other researchers in the Pacific [9,20]. Higher values (48–82%) were found in a large survey in Europe [2,21] and lower values in other countries [22–24], but comparisons should be made cautiously because of the methodological differences between the studies. In this study, boys were more likely to consume EDs than girls. This trend was reported in previous studies [9,19,21]. Similarly, the finding that adolescents from low SES background were more likely to consume EDs lines up with other observations in the Pacific [9] and elsewhere [21,25]. In the non-consumer subgroup, adolescents with high SES were more likely to cite parental refusal of EDC than those with low SES. EDC was also positively correlated with age in the subgroup of boys. Thus, we can hypothesize that parental rules may play a role in EDC depending on SES and age. Parents have been found to influence children's and adolescents’ dietary choices [26–28], and parental monitoring and education may influence ED consumption [19,29]. In a recent study, Costa showed that parents usually discussed the health risks of EDs with adolescents and that this prevented the youngest but not the oldest adolescents from consuming EDs [30]. Moreover, high SES was positively correlated with a poor perception of the ED effect on health. These results suggest that low SES adolescents and their parents must be targeted by prevention programs.

We found a high correlation between the opinions of adolescents concerning the ED effect on health and ED consumption. Our results are in line with those obtained by others [8,31]. Moreover, the perceptions of the ED effect on health were correlated with ethnicity, residence area, weight status, SES and ED consumption. The correlation between ED perception and consumption may have two explanations. First, adolescents who think that EDs are not good for health may simply be less likely to consume EDs. This hypothesis suggests that improving awareness about EDs and their potential health effects among youth may have a positive impact on consumption. Second, the adolescents who regularly consume EDs may justify their choice by saying that EDs are good or neutral for health, even though they might not really think this. Furthermore, EDC is correlated with risky behaviors [5,19]. EDC may be one of these risky behaviors for adolescents, and in this case improving awareness about EDs may not be effective for limiting adolescent ED intake.

Interestingly, Melanesian adolescents and those living in rural areas were more likely to have a good opinion about the ED effect on health, which would explain why EDs were more frequently consumed by these two subgroups. Other studies have also found that EDC may be higher in rural areas [21,25] and in some ethnic subgroups [32–34]. Interestingly, the sugar-sweetened beverage consumption of New Caledonian adolescents was likewise higher in rural than urban environments [13]. School prevention and/or parental monitoring may differ across these subgroups, but this hypothesis needs further investigation.

Previous studies have found that digital marketing was more strongly associated with young adults’ EDC than other types of marketing [35–37], but this trend cannot explain the higher consumption in rural areas, where 55–70% of the adolescents are connected to digital social networks compared with the 88% in the urban area [38]. In the same manner, the availability of and access to EDs might play a role in consumption. However, we did not have specific data about ED availability and accessibility in New Caledonia, and future investigations about the accessibility of healthy versus unhealthy foods in rural/urban areas may therefore be of interest.

Concerning the total amount of EDC, we found a mean value of 2130 mL/week (303 mL/day) in the consumer subgroup. This value is notably high compared with the 70 mL/day (2.1L/month) estimated in a large European survey [2]. Moreover, we found that 74.2% of ED consumers drank 2 cans/week or more, and this value is very high compared with the 26% of adolescents in Europe drinking EDs twice a week or more [2]. In New Zealand, 28% of the Pacific Islanders reported consuming EDs four times or more in the previous week [9], whereas 43.5% reported this quantity in our study. EDs can lead to caffeine intoxication and a recommended daily limit of 2 cans/day has been proposed for adults. Our finding that 11.5% of New Caledonian adolescents reported drinking more than 2 cans/day is a mean value that does not take into account the possibility that EDC may be higher on certain days and zero on others. In Australia, 6.2% adolescents exceeded the recommended daily limit of two standard EDs [20]. Comparisons must nevertheless be made with caution because of the different methods used to obtain the data. Moreover, these data [2,9] were obtained in 2012 in a growing ED market showing worldwide consumption increases. It can still be suggested that the EDC of New Caledonian adolescents is notably high.

The results of this study should be viewed in the light of the potential limitations. First, we determined correlations and not causal relationships because the study design was cross-sectional and not longitudinal. Second, the EDC was evaluated by self-reporting so the consumption may have been over- or under-reported by the participants. Third, the SES indicator was based only on the profession of the head of the household and did not take into account his/her education or income. Last, we did not take into account the availability of EDs although this point might influence consumption.

## Conclusion

High EDC in New Caledonian adolescents is associated with good or neutral perceptions of the ED impact on health. This perception is correlated with SES (low), ethnicity (Melanesian), and area of residence (rural). Moreover, the association between sex (being male) and the total EDC per week is significant. These findings need to be taken into consideration for health education programs in New Caledonia and, more broadly, in the Pacific region. Recently, Francis et al. suggested several intervention strategies to reduce EDC, including policy changes targeting ED sales, packaging, price, and visibility [39]. Future research might examine the feasibility of implementing these interventions in the Pacific area in a school context.

## Supporting information

S1 Table Dataset used in this study. (XLSX)
